# Antegrade common bile duct (CBD) stenting after laparoscopic CBD exploration

**DOI:** 10.4103/0972-9941.37196

**Published:** 2007

**Authors:** Om Tantia

**Affiliations:** Department of Minimal Access Surgery, ILS Multispeciality Clinic, Kolkatta, India

Sir,

We thank Sir A. Cuschieri for interest in our article[1] and appreciate the efforts to give expert opinion and raise few important issues namely

1. Low Negative ERCP rate - 5% (i.e. 16/316 cases)
2. Technique used for CBD clearance 3. Technique used for CBD drainage 4. Indications of Bilio-enteric bypass

Firstly, for the low negative ERCP rate, we would like to clarify that in this series ERCP was attempted in patients who had confirmed CBD calculi on radiological imaging (USG / MRCP) and not just the suspicion on biochemical investigations. As such, the negative ERCP rates are expected to be low but we would stress that even with evidence of CBD stone (on radiological imaging) there was 5% negative ERCP rate.

Secondly, regarding the technique of ductal clearance, we agree that trans-cystic approach is a much better method in terms of reduced morbidity to the patient. However, we did not have an access to fine choledochoscope needed for this procedure. To add further, the stones in this part of the country are too large and fill the entire CBD (as mentioned by Sir A. Cuschieri also) which precludes the trans-cystic exploration

Thirdly, as for CBD drainage we agree with Sir A. Cuschieri and accept both options (endobiliary antegrade stent and cystic duct drainage cannula - Fr 8). We also agree that drainage should be done in cases where choledochotomy and CBDE has been attempted. However, we prefer to stent rather than keeping trans-cystic cannula due to the morbidity of tube attached to the abdominal wall for up to two weeks and it being the potential route for infection. As for additive advantage of obtaining a post-op contrast study with cannula, we do not think it is needed if clearance of CBD has been confirmed by choledochoscopy.

Finally, regarding Biliary Enteric Anastomosis, 59 patients (33%) who had undergone this procedure were mainly concentrated in the early part of our series when we did not have the choledochoscope in our infrastructure and so in cases with dilated CBD (> 15 mm) with a large calculi load where there was slightest suspicion of left out stone or recurrence we preferred doing side-to-side choledocho-duodenostomy since follow-up of patients cannot be guaranteed in this part of the country and they usually end up presenting late with complications. Now, with availability of choledochoscope, we do confirm complete clearance of CBD and so bilio-enteric drainage is rarely needed as indicated by Sir Cuschieri.
